# 4-Hydroxy-2,3-Dimethyl-2-Nonen-4-Olide Has an Inhibitory Effect on Pro-Inflammatory Cytokine Production in CpG-Stimulated Bone Marrow-Derived Dendritic Cells

**DOI:** 10.3390/md14050088

**Published:** 2016-05-04

**Authors:** Zahid Manzoor, Jung-Eun Koo, Irshad Ali, Jung-Eun Kim, Sang-Hee Byeon, Eun-Sook Yoo, Hee-Kyoung Kang, Jin-Won Hyun, Nam-Ho Lee, Young-Sang Koh

**Affiliations:** 1 School of Medicine and Brain Korea 21 PLUS Program, Jeju National University, Jeju 690-756, Korea; whitebrands57@yahoo.com (Z.M.); hohoje@nate.com (J.-E.K.); irshad.qau200@gmail.com (I.A.); eunsyoo@jejunu.ac.kr (E.-S.Y.); pharmkhk@jejunu.ac.kr (H.-K.K.); jinwonh@jejunu.ac.kr (J.-W.H.); 2 Institute of Medical Science, Jeju National University, Jeju 690-756, Korea; 3 Department of Chemistry and Cosmetics, College of Natural Sciences, Jeju National University, Jeju 690-756, Korea; jungeun0615@nate.com (J.-E.K.); saeukkang@jejunu.ac.kr (S.-H.B.); namho@jejunu.ac.kr (N.-H.L.)

**Keywords:** 4-hydroxy-2,3-dimethyl-2-nonen-4-olide, inflammation, activator protein-1, pro-inflammatory cytokine

## Abstract

This study was intended to assess the anti-inflammatory properties of 4-hydroxy-2,3-dimethyl-2-nonen-4-olide (Comp) isolated from *Ulva pertusa* Kjellman on production of pro-inflammatory cytokines. Comp revealed remarkable inhibitory effects on production of pro-inflammatory cytokines in bone marrow-derived dendritic cells (BMDCs). Comp pre-treatment in the CpG DNA-stimulated BMDCs exhibited strong inhibition of interleukin (IL)-12 p40 and IL-6 production with IC_50_ values ranging from 7.57 ± 0.2 to 10.83 ± 0.3, respectively. It revealed an inhibitory effect on the phosphorylation of ERK1/2, JNK1/2, and p38, and on activator protein (AP)-1 reporter activity. Comp displayed noteworthy inhibitory effects on phosphorylation and degradation of IκBα, and on NF-κB reporter activity. In summary, these data propose that Comp has substantial anti-inflammatory properties and warrants further study concerning its potential use as a therapeutic agent for inflammation-associated maladies.

## 1. Introduction

Pathogen-associated molecular patterns (PAMPs) and damage-associated molecular patterns (DAMPs) are recognized by cells of the innate immune system known as pattern recognition receptors (PRRs) [1,2]. Among various PRRs, toll-like receptors (TLR) play an essential role in detecting several types of PAMPS and manage appropriate innate and adaptive immune responses [3].

Dendritic cells are key cellular component of mammalian immune system and known as antigen presenting cells. TLRs are expressed in BMDCs and play vital role in detection of various PAMPs. The main function of BMDCs is to process the antigen and present it to other cells of immune system. Stimulation of BMDCs leads to the production of various pro-inflammatory cytokines, including interleukin (IL)-12 p40, IL-6, and tumor necrosis factor (TNF)-α [4,5,6].

Inflammation is a complex body response of the host against harmful stimuli, including microbial infection, damaged cells, and autoimmune disorders [7]. It protects the body against damage caused by harmful stimuli, helps to eradicate the cause of cell injury, and to initiate the repair of damaged tissue [4].

Recognition of PAMPs by TLR in BMDCs initiates activation of downstream signaling cascades comprising of mitogen-activated protein kinases (MAPKs) and nuclear factor kappa-light-chain enhancer of activated B cells (NF-κB) pathways [8]. Ultimately, activation of MAPKs and NF-κB pathways leads to the production of IL-12 p40, IL-6, and TNF-α and induces an adaptive immune response [2,4,5]. Different TLRs can be activated by their specific ligands. TLR9 is responsible for detection of unmethylated CpG motif present in microbial DNA sequences [9,10]. Nevertheless, mammalian self-DNA incorporated into immune complexes can be responsible for some autoimmune disorders [9,11]. Similarly, TLR4 is responsible for detection of lipopolysaccharide (LPS), a bacterial endotoxin and strong inducer of inflammation [1]. Controlled production of pro-inflammatory cytokines is critical for host defense. However, overproduction of these pro-inflammatory cytokines has been reported to be associated with various disorders [12,13].

MAPKs are a conserved family of serine/threonine protein kinases and play critical roles in various basic cellular processes including proliferation, motility, stress response, survival, and apoptosis [14,15]. The well-characterized subfamilies of MAPKs include the extracellular signal-regulated kinase (ERK), the p38 family of kinases (p38), and the c-Jun N-terminal kinases (JNKs) [15,16]. In mammalian cells, ERK1/2, JNK1/2, and p38α have been well-studied in the milieu of innate immunity [17]. ERK1/2 activation results in phosphorylation of various substrates, such as cytoskeletal proteins, some membrane proteins, and activator protein-1 (AP-1) [18,19]. AP-1 is a transcription factor involved in managing various cellular processes, including proliferation and apoptosis [20]. JNKs are also called as stress-associated kinases and activated by cytokines [16]. All of the MAPKs, in turn, play significant roles in innate immunity [21].

Marine algae are a rich source of highly bioactive secondary metabolites. Marine macro algae have a broad spectrum of biological activities, including antimicrobial, antiallergic, anticancer, and antioxidant activities [22]. During the past few decades, a large number of novel compounds have been isolated from marine organisms and they have interesting biological activities [23]. *Ulva pertusa* Kjellman (*U. pertusa* Kjellman) belongs to the genus *Ulva* in family of *Ulvaceae*. Genus *Ulva* has been extensively studied for biologically-active compounds. The members of the marine green algal genus *Ulva* are used as food for human beings in different parts of world. These are well known for high protein content, soluble dietary fiber, and minerals [24]. Previous studies showed that *U. pertusa* Kjellman has various biological activities, including antimicrobial and anti-inflammatory properties. It has been suggested that *U. pertusa* Kjellman has immunomodulatory function for inflammatory response in broiler chicks [25]. Other members of genus *Ulva*, like *Ulva lactuca* and *Enteromorpha intestinalis* have been shown to possess antioxidant, antimicrobial, and anticancer activities [22]. In addition, *U. fasciata* has been reported to possess anti-inflammatory activity [26]. Previously 4-hydroxy-2,3-dimethyl-2-nonen-4-olide (Comp) has been isolated from *Juncus effusus* and a medium of *Marchantia polymorpha* cell cultures [27,28]. Comp has also been isolated from cress seeding and reported as a plant growth inhibitor [29]. It has been reported that Comp isolated from *Eucheuma cottonii* is helpful in the prevention of hair loss [30]. During continuing research to assess the biological effects, in the present study, Comp was isolated from *U. pertusa* Kjellman and was examined for the anti-inflammatory activities for the first time. Nevertheless, the detailed mechanisms for the anti-inflammatory activities of Comp have not been discussed. Here, we investigated the effect of this compound on primary murine BMDCs and human embryonic kidney cell line 293 T (HEK293T) cells and explored the mechanisms for its observed effects.

## 2. Results

### 2.1. Effects of Comp on the Cell Viability of BMDCs

**Figure 1:**
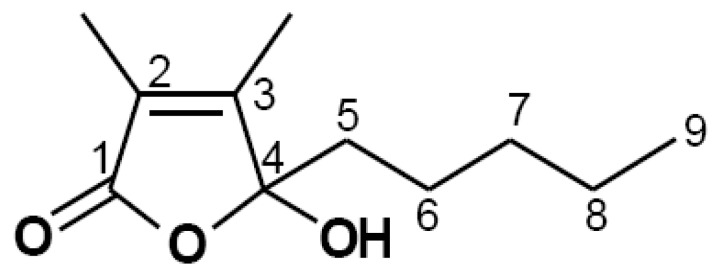
Chemical structure of 4-hydroxy-2,3-dimethyl-2-nonen-4-olide (Comp).

**Figure 2:**
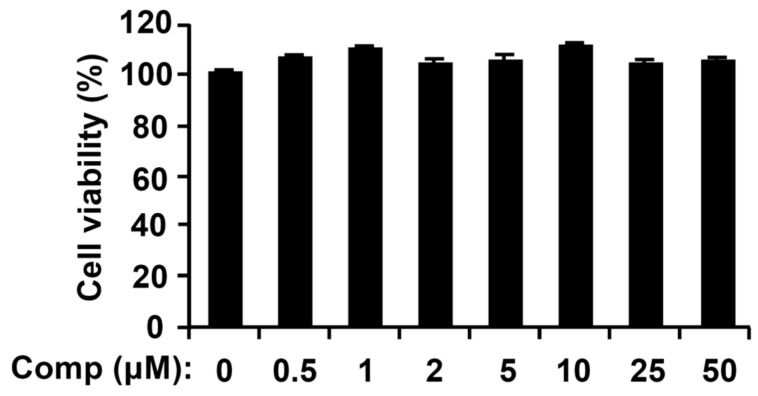
Effects of Comp on cell viability of bone marrow-derived dendritic cells (BMDCs). BMDCs were treated with Comp (1–50 μM) for 18 h and viability was measured using MTT assay. Data are representative of three independent experiments. Comp, 4-hydroxy-2,3-dimethyl-2-nonen-4-olide.

The chemical structure of Comp isolated from *U. pertusa* Kjellman is shown (Figure 1). To assess possible cytotoxicities of Comp on BMDCs, cell viability was determined by using colorimetric 3-(4,5-dimethyl-2,5 thiazolyl)-2,5 diphenyl tetrazolium bromide (MTT) assay. The results showed that viability of BMDCs was not significantly influenced by Comp at the indicated concentrations (Figure 2).

### 2.2. Inhibitory Effects of Comp on IL-12 p40, IL-6, and TNF-α Production in CpG DNA-Stimulated BMDCs

**Figure 3:**
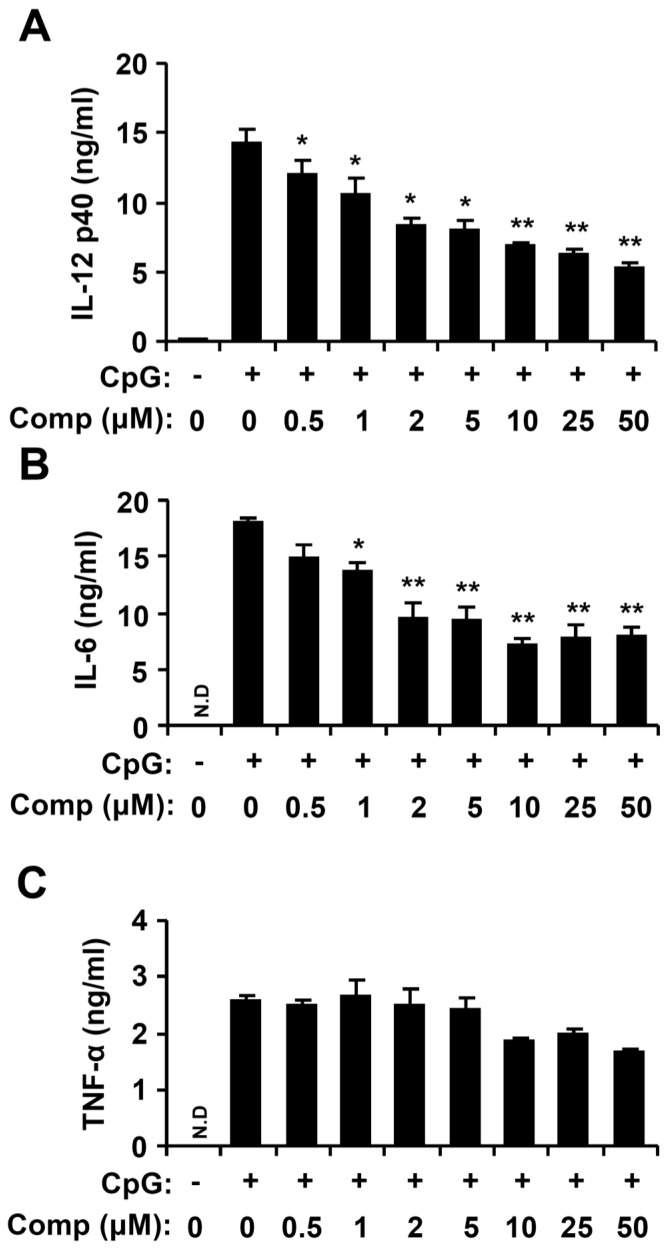
Inhibitory effects of Comp on pro-inflammatory cytokine production in CpG DNA-stimulated bone marrow-derived dendritic cells (BMDCs). BMDCs were treated with Comp at the indicated doses for 1 h before stimulation with CpG DNA (1 μM). Enzyme-linked immunosorbent assay (ELISA) was used to measure the concentrations of murine IL-12 p40 (**A**), IL-6 (**B**), and TNF-α (**C**) in the culture supernatants. Data are representative of three independent experiments. N.D, not detectable; Comp, 4-hydroxy-2,3-dimethyl-2-nonen-4-olide. * *p* < 0.05, ** *p* < 0.01 *vs.* Comp-untreated cells in the presence of CpG DNA.

Dendritic cells have a foremost role in the key cytokines production, including IL-12 p40, IL-6, and TNF-α [4,31]. To explore Comp for anti-inflammatory activity, Comp was tested for inhibitory effects on CpG DNA-stimulated cytokine production in BMDCs. Comp treatment alone showed no production of cytokines (data not shown). CpG DNA induced a substantial increase of IL-12 p40, IL-6, and TNF-α production in BMDCs. Comp pre-treatment intensely inhibited IL-12 p40 and IL-6 production in the CpG DNA-stimulated BMDCs with IC_50_ values of 7.57 ± 0.2 and 10.83 ± 0.3 μM, respectively (Figure 3). However, it did not show significant inhibition on TNF-α production (IC_50_ > 100 μM, Figure 3). Together, these data demonstrate that Comp had an inhibitory effect on IL-12 p40 and IL-6 production in CpG DNA-stimulated BMDCs.

### 2.3. Effects of Comp on the Phosphorylation of MAPK by CpG-Stimulated BMDCs

**Figure 4:**
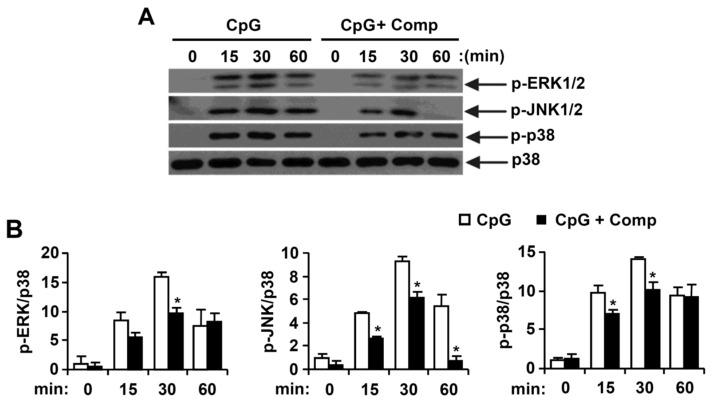
Effects of Comp on the phosphorylation of MAPK by CpG DNA-stimulated BMDCs. (**A**) Cells were pre-treated with or without Comp (25 µM) for 1 h before stimulation with CpG DNA (1 µM). Total cell lysate was obtained at the indicated time intervals. Western blot analysis was performed on the cell lysate to assess phosphorylation of ERK, JNK, and p38. Total p38 MAPK was taken as the loading control. Data are representative of three independent experiments; and (**B**) phosphorylation of ERK, JNK, and p38 protein expression was quantified using scanning densitometry, and the band intensities were normalized by that of total p38 protein. Comp, 4-hydroxy-2,3-dimethyl-2-nonen-4-olide. * *p* < 0.05 *vs.* Comp-untreated cells in the presence of CpG DNA.

CpG DNA triggers the stimulation of TLR9 which, in turn, activates downstream NF-κB and MAPK pathways and ultimately results in pro-inflammatory cytokines production [4,32]. Thus, MAPK phosphorylation was examined in CpG DNA-stimulated BMDCs, with or without Comp treatment by Western blot analysis (Figure 4). Stimulation of BMDCs with CpG DNA resulted in phosphorylation of all three MAPKs (Figure 4). Phosphorylation of ERK1/2, JNK1/2 and p38 was observed between 15 to 60 min of CpG DNA-stimulation. Comp pre-treatment in the presence of CpG DNA revealed strong inhibition of ERK1/2 and JNK1/2 phosphorylation (Figure 4A,B). It also showed inhibition of p38 phosphorylation at 15 and 30 min of stimulation (Figure 4A,B). Together, these data suggest that Comp can inhibit CpG-stimulated ERK1/2, JNK1/2, and p38 phosphorylation in BMDCs.

### 2.4. Effects of Comp on the Phosphorylation and Degradation of IκBα by CpG-Stimulated BMDCs

**Figure 5:**
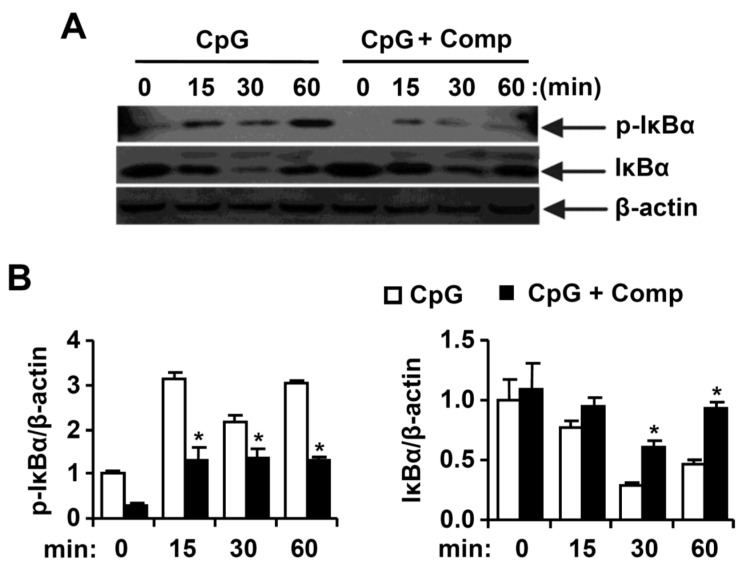
Effects of Comp on the phosphorylation and degradation of IκBα by CpG DNA-stimulated BMDCs. (**A**) Cells were pre-treated with or without Comp (25 µM) for 1 h before stimulation with CpG DNA (1 µM). Total cell lysate was obtained at the indicated time intervals. Western blot analysis was performed on the cell lysate to assess the phosphorylation and degradation of IκBα. β-actin was taken as the loading control. Data are representative of three independent experiments; and (**B**) phosphorylation and degradation of IκBα protein expression was quantified using scanning densitometry, and the band intensities were normalized by that of β-actin protein. Comp, 4-hydroxy-2,3-dimethyl-2-nonen-4-olide. * *p* < 0.05 *vs.* Comp-untreated cells in the presence of CpG DNA.

Stimulation of TLR leads to phosphorylation of IκB by IκB kinase, and the subsequent ubiquitination and degradation of IκB results in activation of NF-κB [4]. Stimulation of BMDCs with CpG DNA resulted in phosphorylation of IκBα (Figure 5). Comp pre-treatment in the presence of CpG DNA revealed strong inhibition of IκBα phosphorylation (Figure 5A,B). Activation of NF-κB was also assessed indirectly by the degradation of IκBα. CpG DNA-stimulation induced IκBα degradation within 30 min of stimulation (Figure 5A,B). The amount of IκBα protein returned to baseline levels after 60 min post-stimulation. Comp pre-treatment inhibited IκBα degradation and, hence, the activation of NF-κB in CpG DNA-stimulated BMDCs (Figure 5A,B). Taken together, these data suggest that Comp inhibited the NF-κB activation.

### 2.5. Comp Treatment Inhibited AP-1 Reporter Activity in HEK293T Cells

**Figure 6:**
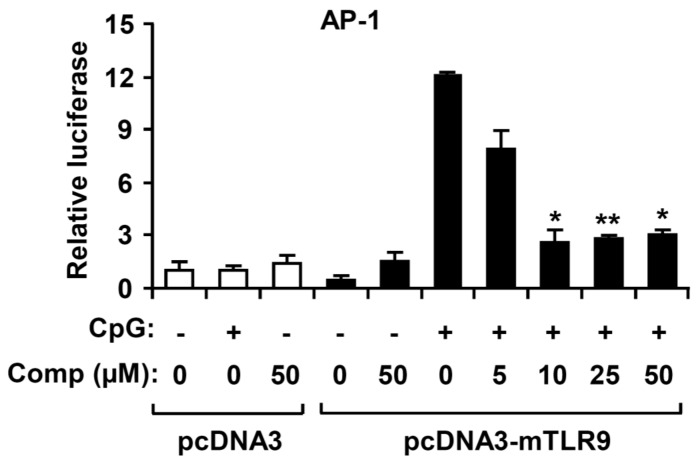
Effects of Comp on AP-1 reporter activity in HEK293T cells. HEK293T cells were transfected with an empty vector (pcDNA3) or a murine TLR9-expressing plasmid (pcDNA3-mTLR9) and then treated with Comp for 1 h before stimulation with CpG DNA (1 μM). Cell lysates were prepared, luciferase activity was assayed by the dual luciferase reporter assay, and the results were expressed as relative luciferase. Data are representative of three independent experiments. Comp, 4-hydroxy-2,3-dimethyl-2-nonen-4-olide. * *p* < 0.05, ** *p <* 0.01 *vs.* Comp-untreated cells in the presence of CpG DNA.

AP-1 transcriptional activity is increased upon activation of MAPK, which ultimately results in expression of multiple AP-1-regulated genes, including pro-inflammatory cytokines [1,32]. To explore whether the Comp had an inhibitory effect on CpG DNA-stimulated AP-1 transcriptional activity, an AP-1 reporter gene assay was performed (Figure 6). The HEK293T cells transfected with empty pcDNA3 showed little AP-1-dependent luciferase activity on CpG DNA stimulation. In contrast, the HEK293T cells transfected with pcDNA3-mTLR9 exhibited robust AP-1-dependent luciferase activity on CpG DNA stimulation. Comp pre-treatment showed strong inhibition of AP-1-dependent luciferase activity in HEK293T cells transfected with pcDNA3-mTLR9 (Figure 6). Hence, this data suggest that Comp has an inhibitory effect on TLR9-dependent AP-1 activation on CpG DNA stimulation.

### 2.6. Comp Treatment Inhibited NF-κB Reporter Activity in HEK293T Cells

**Figure 7:**
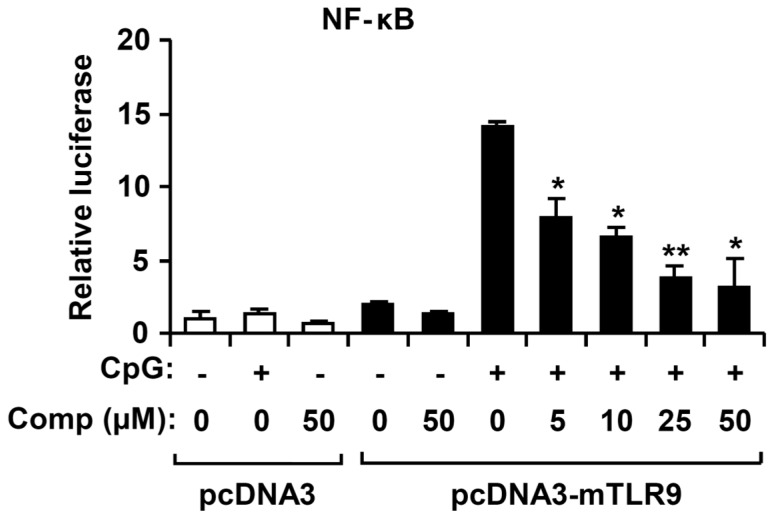
Effects of Comp on NF-κB reporter activity in HECK293T cells. HEK293T cells were transfected with an empty vector (pcDNA3) or a murine TLR9-expressing plasmid (pcDNA3-mTLR9) and then treated with Comp for 1 h before stimulation with CpG DNA (1 μM). Cell lysates were prepared, luciferase activity was assayed by the dual luciferase reporter assay, and the results were expressed as relative luciferase. Data are representative of three independent experiments. Comp, 4-hydroxy-2,3-dimethyl-2-nonen-4-olide. * *p* < 0.05, ** *p* < 0.01 *vs.* Comp-untreated cells in the presence of CpG DNA.

Activation of the NF-κB pathway results in nuclear translocation and binding of this transcriptional factor to its target promoter sites [1]. To examine whether Comp has an inhibitory effect on CpG DNA-stimulated NF-κB transcriptional activity, an NF-κB reporter gene assay was conducted (Figure 7). The HEK293T cells transfected with empty pcDNA3 exhibited no NF-κB-dependent luciferase activity on CpG DNA stimulation. The HEK293T cells transfected with pcDNA3-mTLR9 revealed robust NF-κB-dependent luciferase activity on CpG DNA stimulation. However, Comp pre-treatment exhibited strong dose dependent inhibition of NF-κB-dependent luciferase activity in HEK293T cells transfected with pcDNA3-mTLR9 (Figure 7). Therefore, this data suggest that Comp has an inhibitory effect on TLR9-dependent NF-κB activation on CpG DNA stimulation.

### 2.7. Effects of Comp on the Phosphorylation of MAPK and IκBα, and Degradation of IκBα by LPS-Stimulated BMDCs

**Figure 8:**
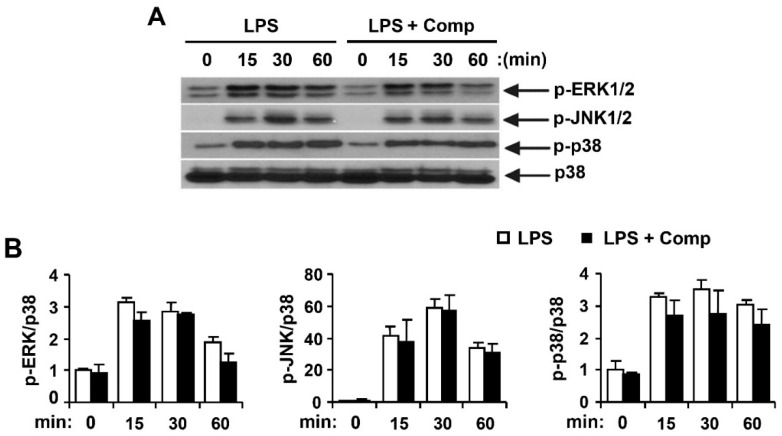
Effects of Comp on the phosphorylation of MAPK by LPS-stimulated BMDCs. (**A**) Cells were pre-treated with or without Comp (25 µM) for 1 h before stimulation with LPS (10 ng/mL). Total cell lysate was obtained at the indicated time intervals. Western blot analysis was performed on the cell lysate to assess phosphorylation of ERK, JNK and p38. Total p38 MAPK was taken as the loading control. Data are representative of three independent experiments; and (**B**) phosphorylation of ERK, JNK, and p38 protein expression was quantified using scanning densitometry, and the band intensities were normalized by that of total p38 protein. Comp, 4-hydroxy-2,3-dimethyl-2-nonen-4-olide.

**Figure 9:**
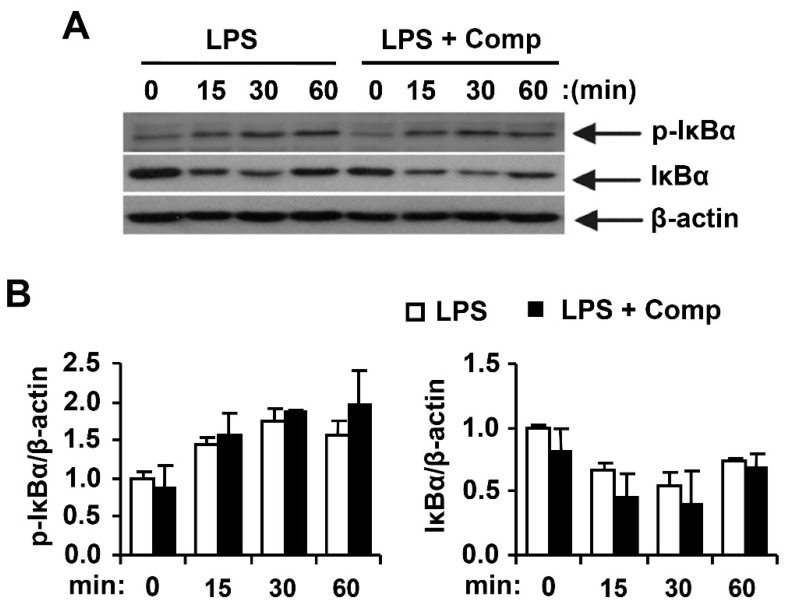
Effects of Comp on the phosphorylation and degradation of IκBα by LPS-stimulated BMDCs. (**A**) Cells were pre-treated with or without Comp (25 µM) for 1 h before stimulation with LPS (10 ng/mL). Total cell lysate was obtained at the indicated time intervals. Western blot analysis was performed on the cell lysate to assess the phosphorylation and degradation of IκBα. β-actin was taken as the loading control. Data are representative of three independent experiments; and (**B**) phosphorylation and degradation of IκBα protein expression was quantified using scanning densitometry, and the band intensities were normalized by that of β-actin protein. Comp, 4-hydroxy-2,3-dimethyl-2-nonen-4-olide.

Activated MAPK and NF-κB pathways by TLR4 result in the production and release of pro-inflammatory cytokines [4]. To understand whether Comp blocks a specific pathway downstream of TLR9 or has a more general anti-inflammatory effect mediated by other TLRs such as TLR4, we studied the effects of Comp on phosphorylation of MAPK and NF-κB activation in LPS-stimulated BMDCs by Western blot analysis (Figure 8 and Figure 9). LPS stimulation of BMDCs significantly increased the phosphorylation of all three MAPKs; ERK1/2, JNK1/2, and p38 (Figure 8). However, Comp pre-treatment did not show inhibition of ERK1/2, JNK1/2, and p38 phosphorylation (Figure 8). 

NF-κB is a critical transcription factor involved in the expression of different cytokines [1]. Stimulation of BMDCs with LPS resulted in phosphorylation of IκBα (Figure 9). Comp pre-treatment in the presence of LPS showed no inhibition on phosphorylation of IκBα (Figure 9). Activation of NF-κB was also assessed indirectly by the degradation of IκBα. LPS-stimulation induced IκBα degradation within 15 min of stimulation (Figure 9). However, Comp pre-treatment showed no inhibition on IκBα degradation and, therefore, no blockage of NF-κB activation in LPS-stimulated BMDCs (Figure 9). Together, these data suggest that Comp does not block the TLR4-specific pathway.

## 3. Discussion

In this study, the anti-inflammatory effects of Comp, an ingredient of *U. pertusa* Kjellman, was investigated. To the best of our knowledge, this is the first report to establish that Comp isolated from *U. pertusa* Kjellman has the potential for medicinal solicitation in inflammation-associated diseases.

BMDCs play pivotal roles in host defense by producing pro-inflammatory cytokines, including IL-12 p40, IL-6, and TNF-α. IL-12 p40 is an important cytokine in Th1-mediated autoimmune responses and involved in critical immunoregulatory activities. Therefore, down regulation of IL-12 p40 production by Comp may have beneficial effects in fighting against IL-12 p40-related autoimmune diseases [8,33]. IL-6 is a pleiotropic cytokine and play vital roles in inflammation, hematopoiesis, regulation of cell growth, and differentiation [34,35]. As cancer is basically a disease of tissue growth regulation failure, IL-6 has important roles in the pathophysiology of cancer. Upregulation of IL-6 has been reported in breast cancer and increased serum IL-6 levels are observed in advanced breast tumor stages [36]. IL-6 has also been associated to various other diseases, including atherosclerosis, depression, diabetes, and rheumatoid arthritis [37]. In this study, pre-treatment of Comp revealed a strong inhibition of IL-6 production in CpG DNA-stimulated BMDCs. Thus, potent inhibitory activity of this Comp against IL-6 production warrants further research concerning potential uses of Comp for anti-inflammatory diseases and inflammation-associated cancer.

The engagement of TLR9 by CpG DNA results in downstream activation of signaling cascades including MAPKs and NF-κB pathways and, ultimately, leads to the production of inflammatory cytokines [38,39]. In the present study, pre-treatment of Comp showed profound inhibitory effects on the phosphorylation of ERK1/2, JNK1/2, and AP-1 reporter activity. It also showed an inhibitory effect on phosphorylation of p38. Comp pre-treatment also strongly inhibited NF-κB activation. Activation of MAPKs and NF-κB pathways is required for transcription of various regulatory genes in the nucleus, including pro-inflammatory cytokines [40]. Moreover, our result from NF-κB-dependent reporter gene assay also revealed that Comp inhibited NF-κB-dependent luciferase activity in HEK293T cells. In the present study Comp did not show an inhibitory effect on phosphorylation of MAPKs or activation of NF-κB in LPS-stimulated BMDCs, suggesting that this Comp has no TLR4-mediated anti-inflammatory activity. Together, these results propose that the inhibitory effect of Comp on pro-inflammatory cytokines production may correlate with inhibition of both MAPKs and NF-κB-dependent pathways which are mediated by TLR9. Thus, Comp-mediated anti-inflammatory activity denotes a potential therapeutic use of compound for inflammation-associated diseases.

## 4. Experimental Section

### 4.1. Isolation of 4-Hydroxy-2,3-dimethyl-2-nonen-4-olide (Comp) from *U. pertusa* Kjellman

Solvent extraction of dried *U. pertusa* Kjellman with 70% aqueous ethanol followed by fraction extract led to n-hexane, ethyl acetate (EtOAc), *n*-butanol and water-soluble portions. A part of the EtOAc-soluble fraction (4.5 g) was subjected to medium pressure liquid chromatography (MPLC) through a reserved-phase silica gel using water-methanol gradients to give 45 fractions. Fraction 22 (80.0 mg) was purified by silica gel with *n*-Hex/EtOAc = 1/1 to give 4-hydroxy-2,3-dimethyl-2-nonen-4-olide (10.1 mg). The structure of the known compound was identified by spectroscopic studies and by comparing the obtained data with literature values [28]. Comp was dissolved in DMSO (Amresco, Solon, OH, USA).

### 4.2. Mice

Six-week-old female C57BL/6 mice were purchased from Orient Bio Inc. (Seongnam, Korea) and maintained under specific pathogen-free conditions. All mice were maintained and used in accordance with institutional and National Institutes of Health guidelines. All animal procedures were approved by and performed according to the guidelines of the Institutional Animal Care and Use Committee of Jeju National University, Jeju, Korea (#2010-0028).

### 4.3. Cell Cultures and Measurement of Cytokine Production

To grow BMDCs, wild-type six-week-old female C57BL/6J mice were used as previously described [41]. Briefly, bone marrow from the tibia and femur was obtained by flushing with DMEM (Gibco, NY, USA), and bone marrow cells were cultured in RPMI 1640 (Gibco, NY, USA) medium containing 20% heat-inactivated FBS, 3% granulocyte-macrophage colony-stimulating factor, and 1% penicillin-streptomycin (Gibco, NY, USA) for dendritic cell generation. For BMDCs, on day 6 of incubation, the cells were harvested and seeded in 48-well plates at a density of 1 × 10^5^ cells/0.5 mL and, then, treated with Comp for 1 h before stimulation with CpG DNA (1 μM). Supernatants were harvested 18 h after stimulation. The concentrations of murine IL-12 p40, IL-6, and TNF-α in the culture supernatants were determined by enzyme-linked immunosorbent assay (ELISA) (BD PharMingen, San Jose, CA, USA, R and D system, MN, USA), according to the manufacturer’s instructions.

### 4.4. Cell Viability Assay

To evaluate cell viability, the standard procedure of 3-(4,5-dimethyl-2,5 thiazolyl)-2,5 diphenyl tetrazolium bromide (MTT) assay was conducted. Briefly, the cells at a concentration of 4 × 10^4^ cells were seeded on a 96-well culture plate. After incubation for 1 h at 37 °C, cells were treated with Comp at different concentrations for 18 h. Cells were added to 0.2 mg MTT (Sigma, St. Louis, MO, USA) and then incubated for 4 h at 37 °C. The plate was centrifuged, and the supernatants were aspirated. The formazan crystals in each well were dissolved in 250 μL dimethyl sulfoxide (DMSO) (Amresco, Solon, OH, USA). Absorbance was measured at a wavelength of 540 nm.

### 4.5. Western Blot Analysis

Western blot analysis was executed using standard techniques as previously described [42,43]. Briefly, BMDCs were dispensed to 35-mm culture dishes (Nunc, Roskilde, Denmark) at 2.5 × 10^6^ cells per dish and incubated for 1 h at 37 °C. The cells were pre-treated with or without Comp (25 µM) for 1 h before treatment with CpG DNA or LPS at the indicated time points. The cells were collected and then lysed in lysis buffer (PRO-PREP lysis buffer, iNtRON Biotechnology, South Korea). A protein sample (30 µg) was subjected to electrophoresis in 10% SDS-polyacrylamide gels and transferred to a polyvinylidene fluoride membrane (Bio-Rad, Hercules, CA, USA). The membrane was incubated with 1/1000-diluted rabbit polyclonal antibodies that specifically recognize phospho-p44/42 (P-ERK1/2), p44/42 MAPK, phospho-p38, p38 MAPK and phospho-SAPK/JNK, SAPK/JNK, IκBα (Cell Signaling Technology, Danvers, MA, USA), and β-actin (Santa Cruz Biotechnology, Santa Cruz, CA, USA). After washing, the membrane was incubated with a horseradish peroxidase-linked goat anti-rabbit IgG (Cell Signaling Technology), and immunoactive bands were detected as previously described [44].

### 4.6. Luciferase Assay

For AP-1 and NF-κB reporter assays, HEK293T cells were plated in 24-well plates and grown overnight as previously described [45]. AP-1 and NF-κB reporters and the pRLnull plasmid used in the luciferase assay were the kind gift of Dr. K. Kobayashi. Murine TLR9 expressing plasmid (pcDNA3-mTLR9) was similarly provided by Dr. R. Medzhitov. HEK293T cells were transfected with AP-1 or the NF-κB reporter gene together with pRLnull and pcDNA3-mTLR9 using Fugene 6 (Roche Diagnostics GmbH, Mannheim, Germany). Cells were then further incubated for 24 h and then pre-treated with Comp for 1 h before stimulation with CpG DNA (1 μM). After further incubation for 18 h, cells were lysed in a passive lysis buffer (Promega, Madison, USA), and firefly luciferase *versus* Renilla activities were measured using a dual luciferase reporter assay system (Promega, Madison, WI, USA).

### 4.7. Data Analysis

All experiments were performed at least three times, and the data are presented as the mean ± the standard deviation (SD) of three independent experiments. One-way ANOVA was used for comparison between the treated and the control groups. *p* < 0.05 was considered statistically significant.

## 5. Conclusions

In conclusion, our results suggest that this compound can act as potent anti-inflammatory drug. It remarkably suppressed multiple inflammatory responses, including pro-inflammatory cytokine production and phosphorylation of MAPK and NF-κB in CpG-stimulated BMDCs. Inhibition of pro-inflammatory cytokines was achieved by attenuating TLR9-dependent AP-1 and NF-κB activation. Based on these results, we conclude that Comp can be developed as an anti-inflammatory drug which might be beneficial in treating inflammation- and autoimmune-associated diseases. Hence, further studies are essential on the detailed mode of action and *in vivo* efficacy of Comp.

## Abbreviations

AP-1, activator protein-1; BMDCs, bone marrow-derived dendritic cells; IC_50_, half maximal inhibitory concentration; N.D., not detectable; DMSO, dimethyl sulfoxide; RPMI, Roswell park memorial institute medium; SDS, sodium dodecyl sulfate; FBS, fetal bovine serum; MAPKs, mitogen activated protein kinases; TLR, toll-like receptors; TNF, tumor necrosis factor; PAMPs, pathogen-associated molecular patterns; DAMPs, damage-associated molecular patterns; PRRs, pattern recognition receptors; IL, interleukin; ERK, extracellular signal-regulated kinase; JNKs, c-Jun N-terminal kinases; NF-κB, nuclear factor kappa-light-chain enhancer of activated B cells; Comp, 4-hydroxy-2,3-dimethyl-2-nonen-4-olide.
